# Cerebral Targeting of Acupuncture at Combined Acupoints in Treating Essential Hypertension: An Rs-fMRI Study and Curative Effect Evidence

**DOI:** 10.1155/2016/5392954

**Published:** 2016-11-28

**Authors:** Yanjie Wang, Yu Zheng, Shanshan Qu, Jiping Zhang, Zheng Zhong, Jialing Zhang, Huanlin Huang, Miaokeng Li, Yiwen Xu, Junqi Chen, Lei Wang, Genevieve Zara Steiner, Chunzhi Tang, Yong Huang

**Affiliations:** ^1^School of Traditional Chinese Medicine, Southern Medical University, Guangzhou, Guangdong Province 510515, China; ^2^Weinan Vocational and Technical College, Weinan, Shaanxi 714026, China; ^3^The Third Affiliated Hospital of Southern Medical University, Guangzhou, Guangdong Province 510630, China; ^4^Weinan Orthopaedics Hospital, Weinan, Shaanxi 714000, China; ^5^The National Institute of Complementary Medicine, School of Science and Health, Western Sydney University, Penrith, NSW 2751, Australia; ^6^Clinical School of Acupuncture and Rehabilitation, Guangzhou University of Chinese Medicine, Guangzhou, Guangdong Province 510405, China

## Abstract

The study attempted to explore that the synergistic effect of acupoints combination is not a simple superposition of single acupoint's effect by comparing and analyzing the changes of blood pressure (BP), SF-36, and brain regions after acupuncture treatment. 47 patients were randomly divided into LR3+KI3 group, LR3 group, and KI3 group. Subjects received Rs-fMRI scan, BP measurement, and SF-36 questionnaires before and after treatment and short-term acupuncture treatment. After treatment, there were no significant differences in BP and SF-36 among 3 groups, compared to the case before treatment, SBP of 3 groups decreased, and DBP significantly decreased while vitality and mental health significantly increased in LR3+KI3 group. Both number and scopes of changes of brain regions in LR3+KI3 group were the largest, which mainly included BAs 3, 4, 8, 19, 21, 24, 32, 44, and 45. In conclusion, acupuncture at LR3+KI3 may auxiliarily reduce BP and improve the vitality and mental health of patients, and the changes of brain regions were related to somatesthesia, movement, vision, audition, emotion and mood, language, memory, etc. BAs 4, 9, 10, 24, 31, 32, and 46 may be the targeting brain areas of acupuncture in assisting hypotension. It is suggested that acupoints combination of LR3+KI3 maybe generates a synergistic effect, and it is not simple sum of single acupoint effect.

## 1. Introduction

Since 1972, one of the long history periods of traditional Chinese medicine therapies, acupuncture began to be applied in the United States and Europe [1]. Nowadays, acupuncture is one of the most popular treatments in alternative medicine; it is increasingly valued by people and widely used in the clinic [2]. Relevant scholar generally recognizes that acupuncture therapy is based on acupoints; two or more than two combined acupoints are used for treating the disease [3, 4], and previous research confirmed that the reasonable acupoints combination mostly played a synergistic effect [5, 6].

Currently, the domestic and foreign research aims to explore the central mechanism of acupuncture therapeutic effects by using a noninvasive, visual, high-resolution, and reproducible functional magnetic resonance imaging (fMRI) technology combined with acupuncture to observe the signal changes of the brain areas caused by acupuncture. However, in most of the previous studies, subjects were mostly healthy people, the observation of single acupoint's effects, comparing effects of single acupoint with nonacupoint, effective acupoint with invalid acupoint, between acupoints, or real acupuncture with sham [7–12]. Recently, fMRI has gradually been applied to study the mechanism of acupuncture therapeutic effects based on some diseases; the study of effects of long-term acupuncture treatment in migraine patients compared the differences in brain activities evocation by active acupoints and inactive acupoints [13]. To study the influence of acupuncture at Waiguan (TE5) on the functional connectivity of the central nervous system of patients with ischemic stroke, Chen et al. [14] compared the differences of regions of interest (ROI) between TE5 acupuncture and nonacupoint acupuncture, as well as verum acupuncture and sham acupuncture. Shi et al. [15] researched an experimental acute low back pain model using fMRI to explore the neural mechanisms of acupuncture analgesia by means of acupuncture stimulation and tactile stimulation at BL40. These studies have revealed the possible mechanism of acupuncture therapeutic effects to some extent, but the clinical effect of acupuncture at two or more acupoints combination and the corresponding change of brain areas is or is not the sum of the single acupoint effects, which needs further study.

Previous acupuncture research based on Block Design fMRI [7, 9, 14] ignored aftereffect of acupuncture and could not completely copy the clinical acupuncture treatment process. At present, resting-state functional magnetic resonance imaging (Rs-fMRI) technology has been gradually applied in the acupuncture research field. In order to avoid the “stress” effect of acupuncture in the course of study, Rs-fMRI scan was conducted after withdrawing the needle, instead of during needle-retaining [8, 16, 17], which makes up for the inadequacies of Block Design and can actually simulate the acupuncture treatment process.

In Rs-fMRI study, regional homogeneity (ReHo) is a popular method of image data analysis. ReHo is a method to assess resting-state brain activity by using the functional coherence of a given voxel with those of its nearest neighbors; the advantage is that it can detect unknown hemodynamic responses that the task driving method can not discover [18]. Therefore, Rs-fMRI technology was used to collect the resting-state brain functional images of subjects before and after acupuncture treatment, and the ReHo was used to analyze the image data in this study.

Essential hypertension (EH) is an important risk factor of various cardiovascular and cerebrovascular diseases, and it has become one of the important challenges of global public-health problem [19]. So far, antihypertensive medication is still dominantly used for treating hypertension; however, due to the side effects or safety issues, such as drug resistance which could affect therapeutic efficacy, this therapy is far from satisfactory, so acupuncture therapy was selected as a commonly used supplementary therapy for hypertension treatment [20]. A systematic review of the current clinical evidence of acupuncture treating hypertension showed that there was some evidence and suggested that there was potential effectiveness [21]; a RCT of acupuncture affects the circadian rhythm of BP suggesting that acupuncture treatment could be useful for improving the circadian rhythm of BP in patients [22]; however, another meta-analysis provided evidence of acupuncture efficacy as an adjunctive therapy to western medicine for treating hypertension, while the evidence for acupuncture alone in lowering BP is insufficient [23]. Yin et al. [24] used acupuncture combined with breathing and easy-walking exercises to treat hypertension, finding that systolic blood pressure (SBP) of patients was significantly decreased after 8 weeks of treatment. Based on the research above, we speculate that acupuncture therapy may have assistant antihypertensive effect.

LR3 was one of the most frequently used acupoints for acupuncture treating hypertension [22, 23, 25]. KI3 was also used for treating hypertension [23]; besides, combination of LR3 and KI3 was often used to treat hypertension clinically [25, 26]. In recent years, research of LR3 and KI3 acupuncture by fMRI is constantly ongoing [9, 27]; our team also carried out Rs-fMRI research of acupuncture combination of LR3 and KI3 [28, 29], single LR3 [30], and single KI3 [31], but all subjects were healthy humans. Therefore, this study carried Rs-fMRI research of acupuncture combination of LR3 and KI3, single LR3, and single KI3, with EH patients.

In the current study, a randomized controlled trial, changes of BP and 36-item Short Form quality of life (SF-36) between baseline and after acupuncture treatment were observed, and Rs-fMRI were adopted to compare the difference in activation of brain regions by acupoint combination (LR3+KI3) and single acupoint (LR3 or KI3) for EH patients. Furthermore, a correlation analysis was performed to investigate the possible correlation among clinical effects, activation of brain regions, and acupoints combination.

In summary, we hypothesize that the acupuncture at LR3+KI3 for treating hypertension can produce synergistic effect; compared with acupuncture at LR3 or KI3, the synergistic effects will be more effective to improve the patients' BP and the change of brain areas of acupoints combination is not a simple superposition of single acupoint.

## 2. Methods

### 2.1. Subjects

150 EH patients in I grade or II grade were recruited from different community hospitals in Guangzhou. This clinical trial was registered in China and approved by the ethical review committee (ChiECRCT-2012011) and clinical trial registry in China (ChiCTR-TRC-12002427).* Inclusion criteria* were as follows: (1) no limitation of gender and being of 35 to 65 years old and right-handed; (2) I stage or II stage and intermediate risk hypertension patients, who met the diagnosis and risk stratification criteria of the World Health Organization (WHO)/International Society of Hypertension (ISH) 2003, and subjects who maintained their original treatment (species and dose of antihypertensive medicine did not change) during the study period and their BP was controlled in the normal range but usually fluctuated (SBP 120~179 mmHg and (or) DBP 80~109 mmHg).* The drop-off criteria* were as follows: (1) hypertensive crisis or other emergencies; (2) realigning and excluding subjects with max head motion > 1.5 mm on any axis and head rotation > 1.5 degrees; (3) inability to complete this study because of personal reasons.* Adverse events* were bleeding, hematoma, fainting, serious pain, local infection, fluctuation of blood pressure, and hypertension crisis. If any adverse events occurred during the study period, all the details were documented in the case report manual.

**Figure 1:**
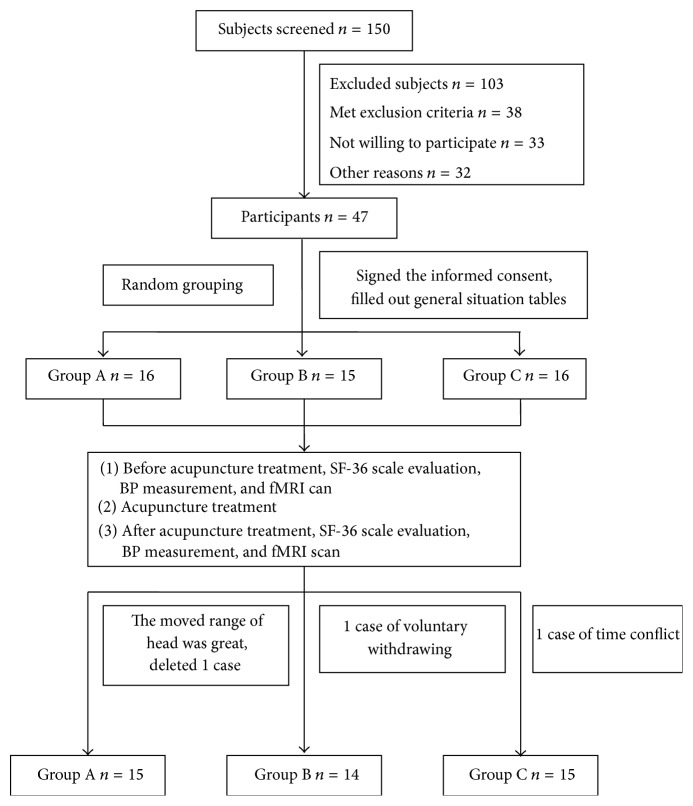
Flowchart of the participants.

According to random numbers table, all subjects were randomly divided into LR3 and KI3 group (group A), LR3 group (group B), and KI3 group (group C) and through the single-blind method (Figure 1). Demographics, including gender, age, heredity, course of disease, SBP, DBP, and SF-36 scale, did not differ among three groups (*P* > 0.05) (see Table 1).

#### 2.1.1. Experimental Procedure

**Figure 2:**
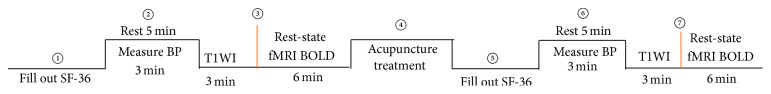
Flowchart of the experimental procedure. T1WI: T1-weighted image; fMRI: functional magnetic resonance imaging; BOLD: blood-oxygen-level dependent contrast; min: minutes.

All subjects filled out SF-36 scale at the beginning of the experiment, and then BP was measured after taking a 5 min rest, and then they were given a baseline fMRI scan. Next, participants of the three groups received acupuncture treatment. All subjects filled out SF-36 scale again the next day after the end of acupuncture treatment, and then BP was measured after taking a 5 min rest, and then they were given the second fMRI scan (Figure 2).

### 2.2. Acupuncture Intervention

**Figure 3:**
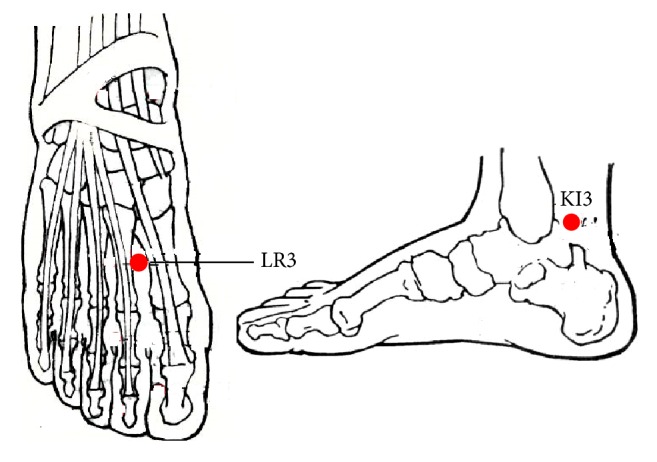
The location of LR3 and KI3.

In group A, acupuncture stimulation was performed at LR3 and KI3. In group B and group C, acupuncture stimulation was performed at LR3 and KI3, respectively. According to the national standards of the location of acupoints (GB/T12346-2006), LR3 locates on the dorsum of the foot, between the first and second metatarsal, in depression of basis metatarsalis junction in front, or touching the arterial pulse. KI3 locates in the ankle area, in the depression between the medial malleolus and achilles tendon (Figure 3).

Acupuncture needles (0.3 mm diameter, 40 mm long, HwaTo, Medical Supplies Co., Ltd., Suzhou) were conventionally inserted. Once sensation of all needles occurred, twisting (90–180°, 60–90 times/min) and lifting-thrusting (0.3–0.5 cm, 60–90 times/min) of each needle were performed for 1 min, and then the needles were retained for 30 minutes; during retaining needles period, the needles were manipulated for 1 minute with an interval of 10 minutes. Acupuncture treatment was carried out once a day, for 5 days, rested for 2 days and then repeated for another 5 days, 10 times in total.

### 2.3. Blood Pressure Measurement

BP measurements were taken before and after the treatment (posttreatment) with mercury sphygmomanometer (Jiangsu Yuyue Medical Equipment Co., Ltd.).

### 2.4. Rs-fMRI Scan

The fMRI scans were carried out in a 3.0 Tesla Signa HDxt MRI scanner (GE Company, Fairfield, America) at First Affiliated Hospital of Guangzhou University of Chinese Medicine. A standard 8-channel phase-array head coil and restraining foam pads were used to minimize head motion.

Subjects were conscious, placed in a supine position, and asked to breathe calmly. Earplugs and special ear shield were used to diminish scanner noise, and eyeshades were used to avoid visual stimulation. During the fMRI scan, subjects were instructed to move as little as possible and if they felt uncomfortable, they should tell investigators loudly and the scan would be stopped. fMRI scan began after subjects resting for 15 min.

MRI data (resting-state BOLD sequence) were collected 15 min before needling and the next day after acupuncture treatment:

1. Transverse T1-weighted image (T1WI) sequence: 1 min, 51 s, Fast Spin Echo sequence; OAx T1 FLAIR, repetition time 1,750 ms/echo time 24 ms, inversion time 960 ms, field of view 24 cm × 24 cm/Z, matrix 320 × 224/number of excitations = 1, thickness 5.0 mm/interval 1.0 mm, 30 slices total, echo train length 8, and bandwidth 31.25.
2. Resting-state fMRI BOLD data collection: gradient echo-echo-planar imaging sequence scanning conducted for 6 min in accordance with the following parameters: repetition time 3,000 ms/minimum, echo time minimum, flip angle 90°, field of view 240 mm × 240 mm.

### 2.5. Statistical Analysis

#### 2.5.1. Image Preprocessing

The preprocessing procedures are the same as those in our previous study [31], which include (1) converting DICOM to NIFTI, (2) slice timing after removing first 10 time points, (3) realigning and excluding subjects with max head motion > 1.5 mm on any axis and head rotation > 1.5 degrees, (4) Coregistering T1 to Fun, (5) segment and affixer regularization according to East Asian, (6) normalizing by using EPI templates, (7) removing linear detrend, and (8) filtering (0.01 Hz–0.1 Hz). However, the data was preprocessed without smoothing.

After preprocessing, 1 case was excluded because the head moving range was too large and 44 cases were included in statistical analysis.

*ReHo Analysis*. Using the REST 1.8 software, KCC map (ReHo maps) was obtained by calculating the Kendall concordance coefficient (KCC value) of the whole-brain voxel of each case; the KCC maps by dividing their whole-brain KCC equalization obtained standardized ReHo value [18].

#### 2.5.2. Data Statistical Analysis

Using the REST 1.8 software, standardized ReHo values of each group's two time points of posttreatment and pretreatment were compared by the paired* t*-test, respectively. In order to avoid the impact of baseline pretreatment, the comparisons of ReHo value among 3 groups before treatment were made using one-way ANOVA; if there was significant difference, ReHo values of 3 groups before treatment would be used as covariates, respectively, the two-independent-sample* t*-test would be used to compare the difference of pretreatment and posttreatment of 3 groups between every two groups [13]. Using xjview 8.0 set statistical threshold probability was 0.05, AlphaSim correction was applied (*P* = 0.05, the cluster size = 228), the changes of ReHo between different groups before treatment and after treatment were obtained, and the brain areas with statistical significance were shown in the form of images. Using SPSS 20.0 for Windows statistical software for statistical analysis of SBP, DBP, and SF-36, the paired* t*-test was used for intragroup comparison of posttreatment and pretreatment; one-way ANOVA or Kruskal-Wallis H was used for comparison among 3 groups.

## 3. Results

### 3.1. The Changes of Average SBP and DBP of the Three Groups

Compared to pretreatment, SBP and DBP of group A obviously reduced after treatment (*P* = 0.002, *P* = 0.038), SBP of group B obviously reduced after treatment (*P* = 0.006), and SBP of group C obviously reduced after treatment (*P* = 0.002). Comparison of three groups' BP values showed that there was no statistical difference after treatment (see Table 2).

### 3.2. The Changes of Average SF-36 Scale of the Three Groups

Compared to baseline, in group A, the SF-36 survey showed that scores of vitality (VT) (mean ± SE, 70.33 ± 4.77 versus 79.33 ± 3.96, *P* = 0.011) and mental health (MH) (mean ± SE, 69.60 ± 4.55 versus 82.40 ± 4.94, *P* = 0.018) improved significantly after acupuncture treatment. Intercomparison of three groups showed that change in MH was significant difference (mean ± SE, group A: 12.80 ± 4.91, group B: −2.57 ± 3.13, group C: 2.27 ± 2.32, *P* = 0.010).

### 3.3. The Results of Rs-fMRI Scan

#### 3.3.1. Changes of Brain Regions of Posttreatment versus Pretreatment in Each Group

**Figure 4:**
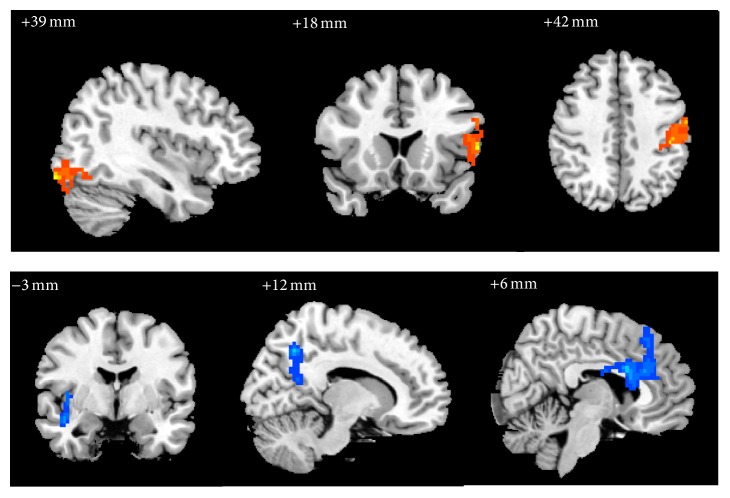
The changes of brain regions of group A after treatment versus before treatment. Note: the orange represents that the ReHo value increased; the blue means the ReHo value decreases.

In group A, compared with pretreatment, the ReHo values of right inferior occipital gyrus (BA19), left inferior frontal gyrus in opercular and triangular part (BAs 44, 45), postcentral gyrus (BAs 3, 4), inferior parietal, excluding supramarginal and angular gyrus (BA3), and left supramarginal gyrus were significantly increased, while right insula, superior temporal gyrus (BA21), precuneus, medial superior frontal gyrus (BA8), anterior cingulate and paracingulate gyrus (BAs 24, 32), and median cingulate and paracingulate gyrus (BA24) were significantly decreased (Table 3, Figure 4).

**Figure 5:**
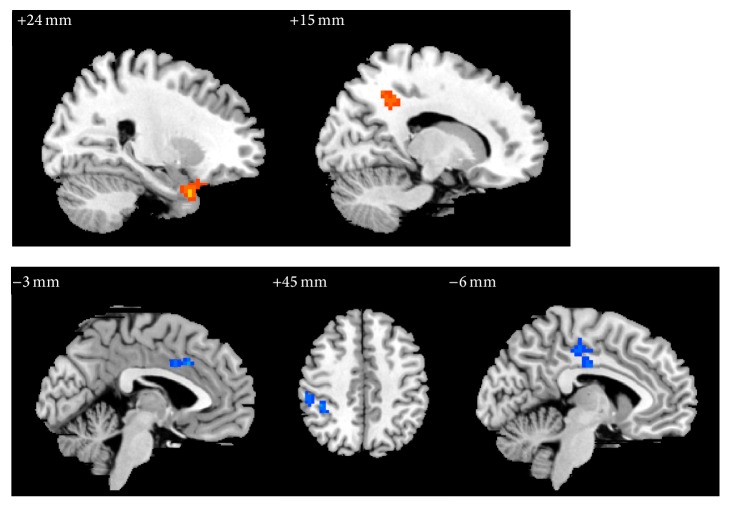
The changes of brain regions of group B after treatment versus before treatment.

In group B, the ReHo values of right hippocampus (BA28), temporal pole in superior and inferior temporal gyrus, precuneus, and BA31 were significantly increased, while the ReHo values of right inferior parietal (excluding supramarginal and angular gyri) (BA40), supramarginal gyrus, left paracentral lobule, and median cingulate and paracingulate gyrus (BA24) were significantly decreased (Table 4, Figure 5).

**Figure 6:**
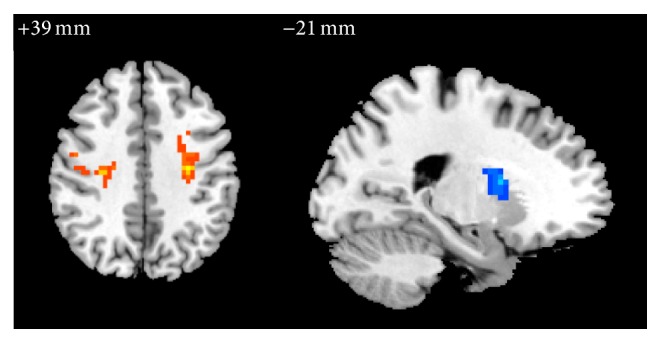
The changes of brain regions of group C after treatment versus before treatment.

In group C, the ReHo values of left putamen and caudate nucleus were significantly increased, while the ReHo values of right postcentral gyrus and left frontal lobe were significantly decreased (Table 5, Figure 6).

#### 3.3.2. The Comparison of Changes of Brain Regions among 3 Groups after Treatment

**Figure 7:**
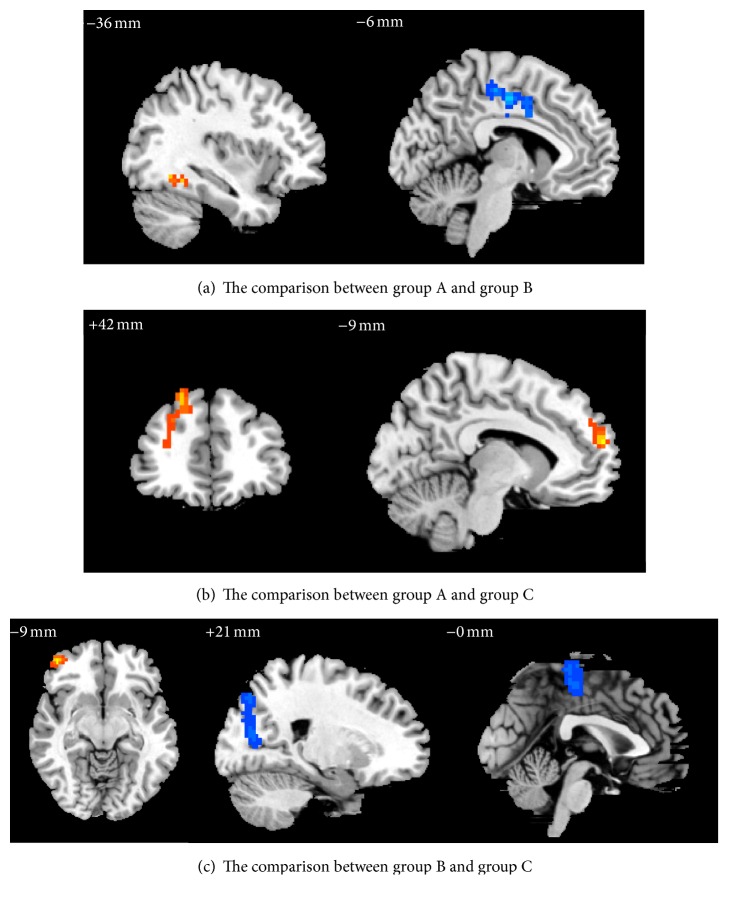
The comparison of changes of brain regions among 3 groups after treatment.

Compared to group B, group A showed that the ReHo values of right precuneus (BA7) and median cingulate and paracingulate gyrus (BA31) were significantly increased, while right postcentral gyrus (BA3) and paracentral lobule (BA5) were significantly decreased (Table 6, Figure 7(a)). Compared to group C, group A showed that the ReHo values of right superior and middle frontal gyrus (BAs 9, 46) and medial superior frontal gyrus (BA10) were significantly increased (Table 6, Figure 7(b)). Compared to C group, B group showed that the ReHo values of right middle frontal gyrus in orbital part (BA46) were significantly increased, while values of right superior and middle occipital gyrus (BAs 18, 19) and paracentral lobule (BA4) were significantly decreased (Table 6, Figure 7(c)).

No serious adverse events happened during the study. Three cases in groups A and B reported having minor hemorrhage at the needling site. They were told to put pressure on the needling areas for 3~5 minutes and recovered in four days.

## 4. Discussion

This is the first Rs-fMRI study that focused on the synergistic effect mechanism of acupoints combination on EH treating by acupuncture. It demonstrated the similarities and differences in SF-36, SBP, and changes of brain regions between acupuncture acupoints combination and single acupoint treatment.

In this study, compared to pretreatment, SBP of three groups posttreatment was significantly reduced, which indicated that acupuncture at acupoints combination (LR3+KI3) or a single acupoint (LR3 or KI3) may adjunctively reduce SBP. In previous studies on acupuncture treatment hypertension, Yin and Du [20] found that pure acupuncture can not only reduce the immediate BP of patients with hypertension, but the hypotensive effect remains stable. However, Macklin et al. [32] found that active acupuncture provided no greater benefit than invasive sham acupuncture in reducing SBP or DBP, and participants were weaned off antihypertensives during the study period. Flachskampf et al. [33] found that in patients who still took antihypertensive drugs according to the original treatment plan during the study period; the BP was significantly lower in the acupuncture group compared with the sham group after 6 weeks of treatment; the change of BP in the subgroup of 35 patients without antihypertensive drugs showed a decreasing trend. Yin et al. [24] applied acupuncture therapy combined with breathing and easy-walking exercises for treating hypertension; SBP of patients was significantly lower after 8-week treatment, which is similar to the results of our study. A study on acupuncture treatment EH indicated that short-term simple acupuncture did not significantly decrease BP, but it may improve quality of life for patients in relation to body pain and vitality [34]. In this study, after acupuncture treatment, some aspects of the quality of EH patients' life have certain influence by acupuncture at LR3+KI3, LR3, or KI3, and acupuncture at acupoints combination can significantly improve VT and MH of subjects; in addition, compared to groups B and C, acupuncture LR3+KI3 can obviously improve MH of EH patients. Both VT and MH are related to subjective feelings; acupuncture at acupoints combination may have some advantages in improving the emotional aspects. Adverse mental stimulation and nervousness of long term have a certain relationship with the occurrence of hypertension. Therefore, this study speculated that antihypertensive mechanism of acupuncture LR3+KI3 may be related to the psychological and emotional regulation. All participants still took antihypertensive medication according to the original therapeutic regimen; SBP of three groups was significantly reduced, so this study prompted that acupuncture therapy may have a certain adjunctive antihypertensive effect. Group A was superior to the other two groups in decreasing DBP and improving MH of hypertension patients; thus, the study infers that acupuncture at LR3+KI3 may produce synergistic effect in the treatment of hypertension.

Rs-fMRI analysis found that there were changed brain areas in the three groups after acupuncture treatment. In group A, the number of both clusters and changes of brain areas was the most; the range of changes of brain areas was also the most changes of brain areas was also the most extensive.

In group A, the changed brain areas relatively concentrated in BAs 3, 4, 8, 19, 21, 24, 32, 44, and 45; the function of the abovementioned brain areas were as follows: (1) somatic sensory cortex, relating to somatic sensory and perception, (2) primary motor cortex, controlling behavior and movement, (3) premotor cortex(area) and frontal eye field, responsible for motion planning and autonomic eye movement, (4) secondary visual cortex, with the function of distinguishing object and face, (5) auditory organization cortex, involving higher auditory processing and language reception, (6) participating in the emotional system and involving the emotional judgment (especially pain) of somatesthesia, motion planning, and memory processing, (7) Broca's area, involving language production, execution of semantics task. In another study on acupuncture at LR3+KI3 of healthy people after withdrawing needles, cerebral blood flow changed in the brain areas relating to vision, emotion and mood, cognition, attention, phonological and semantic processing, memory, and so on [28]; some results of acupuncture at LR3+KI3 after treatment in this study were similar to the abovementioned study; it is suggested that the above brain areas relating to vision, emotion and mood, and language and memory may be somehow stimulated by LR3+KI3.

In group B, the changed brain areas were mostly BAs 24, 28, 31, 38, and 40; the functions of the above brain areas were as follows: (1) the emotional system, involving the emotional judgment (especially pain) of somatesthesia, motion planning, and memory processing, (2) organizing hippocampus allowing the encoding and consolidation of memory, (3) relating to memory and emotion, and (4) secondary somatosensory cortex, responding to somatic stimulation, and facilitating structural differentiation. In acupuncture at LR3 of healthy people after withdrawing needles, the brain areas of changing were related to vision, somatic movement, sensory, emotion and analgesia, and so on [30]; some results of acupuncture at LR3 after treatment in this study were similar to the above study, but there was no significant change in brain areas relating to vision, which was not consistent with the previous studies [35]. It is suggested that above brain areas relating to somatic movement, sensory, emotion, and analgesia may be stimulated by LR3.

The changed brain areas of group C were related to somatesthesia and perception, thinking and planning, individual needs and emotion, and the coordination movement of muscle (maintaining a certain body posture). In acupuncture at KI3 of healthy people after withdrawing needles, the changes of brain areas were related to perception, movement, vision, audition, and spirit [31]; the changes of brain areas of acupuncture at KI3 were related to cognitive function in previous studies [27], while the brain areas relating to vision and audition did not obviously change; the changes of brain areas were only related to perception, movement, and emotion in this study; it is suggested that the above brain areas may be associated with stimulation of KI3.

Some of the changes of brain areas in three groups were related to somatesthesia, movement, emotion, and mood, and some changing brain areas were also related to memory processing in groups A and B; in addition, the changed brain areas of group A were also associated with audition and language. It is suggested that the effect of acupuncture at LR3+KI3 may be based on the effect of single acupoint, and activated brain areas of EH patients with acupuncture at LR3+KI3 are not simple superposition of activated brain areas of acupuncture a single acupoint; namely, that acupoints combination may generate a synergistic effect, which is beyond the simple sum of single acupoint's effect.

The study of hypothalamus-seeded resting brain network underlying short-term acupuncture treatment in primary hypertension found that increased positive correlations were primarily located in the cerebellum, limbic system (insula, parahippocampal gyrus, and cingulate cortex), bilateral thalamus, and frontal lobes after treatment in acupuncture group [34]. In the present study, BP of the three groups decreased after treatment, the changes of brain areas of three groups were located in the frontal lobe, and the changes of brain areas in groups A and B were located in median cingulate and paracingulate gyrus; besides, insula and anterior cingulate changed gyrus in group A, and parahippocampal gyrus changed in group B. Therefore, it is suggested that the above brain areas may be associated with the targeting effect of acupuncture decreasing BP; the brain areas associated with the antihypertensive effect of LR3+KI3 were not simple superposition of the brain areas of targeting effect for antihypertensive effect of LR3 and KI3. Studies confirmed that emotional changes have a certain impact on change of BP; negative emotions have been linked to increases in blood pressure, anxiety, depression, and anger leading to increased risk of hypertension [36], and the findings indicated an association between high positive emotion and lower blood pressure among older Mexican Americans [37]. BAs 24, 31, and 32 are related to emotion; therefore, it is speculated that they may be the brain areas of targeting effect for acupuncture assistant antihypertensive effect.

Compared with group B, the changed brain areas in group A mainly included BAs 3, 5, 7, and 31, function of which are as follows: (1) somatosensory projection cortex and responsibility for somatosensory and perception, (2) somatosensory association cortex and vision-motor coordination, and (3) emotion processing and recognition. Because the emotional change has a certain effect on BP and BA31 is related to emotion processing and recognition, ReHo value of BA31 increased, its consistency of local neuronal activity increased, and connection with the activity of peripheral neurons enhanced; only SBP decreased in group B; both SBP and DBP decreased in group A, so it was further confirmed that BA31 may be one of the brain areas responding to the targeting effect difference of acupuncture LR3+KI3 and single LR3 regulating BP changes.

Compared with group C, the changed brain areas in group A mainly included BAs 9, 10, and 46; they belong to the dorsal lateral prefrontal cortex; their function relates to performance and cognition function, such as working memory and higher cognitive processing, central decision making and performance, and executive function. Compared with group C, the change brain areas in group B mainly included BAs 4, 18, 19, and 46, function of which is as follows: (1) primary motor cortex and controlling behavior motion, (2) vision association cortex and responsibility for visual processing, and (3) executive function. In the study on functional connectivity of acupuncture treating hypertension, functional connectivity between bilateral frontal lobe and hypothalamus positively correlated, and there is a fiber connection between the prefrontal cortex and dorsomedial nucleus; dorsomedial nucleus is the location of complex integration effect of viscus and idiosoma activities. BAs 4, 9, 10, and 46 are located in the frontal lobe, SBP of group B and group C decreased, and both SBP and DBP of group A decreased; therefore, it is speculated that BAs 4, 9, 10, and 46 may be the brain areas responsive to acupuncture's adjunctive antihypertensive effect. The previous research found that acupuncture at LR3 could regulate the frontal lobe (such as BAs 4, 6, 9, 46, etc.) [38]. In both group A and group B compared with group C in this study, BAs 4, 9, and 46 changed; they were located in frontal lobe, and LR3 was needled in two groups; thus, this study further confirmed that acupuncture LR3 can modulate the frontal lobe function.

There are several limitations in this study; for instance, the sample size is small, gender ratio of male and female is imbalanced, antihypertensive drugs are still being taken during the study period, the observation time is short, there is no control group (either waitlist or sham), and there are no long-term follow-up and evaluation of long-term curative effect; the above problems will be improved in the following research.

## 5. Conclusions

This study found that acupuncture at LR3+KI3 has the potential to auxiliarily decrease BP of EH patients, strengthen the effect of antihypertensive medication, and improve the vitality and mental health of patients. After treatment, the number of changed brain areas were the most, and the range was also the most extensive in acupoints combination group; these brain areas were related to somatesthesia, movement, vision, audition, emotion and mood, language, memory, and so forth. It is suggested that the effect of acupuncture at LR3+KI3 may be based on the effect of single acupoint, and activated brain areas of hypertension patients with acupuncture at LR3+KI3 are not simple superposition of activated brain areas of acupuncture a single acupoint; namely, LR3+KI3 acupoints combination may generate a synergistic effect, and it is not simple sum of single acupoint effect. BAs 4, 9, 10, 24, 31, 32, and 46 may be the targeting brain areas of acupuncture in assisting hypotension; however, how they affect blood pressure change is yet to be further studied.

## Figures and Tables

**Table 1:** Baseline characteristics ($\overset{-}{X}\pm SD$ or $\overset{-}{X}\pm SE$).

Characteristic		Group A	Group B	Group C	Statistical value	*P*
Gender (%)						
Male		5 (33.33)	1 (7.10)	5 (33.33)	*χ* ^2^ = 3.492	0.174
Female		10 (66.67)	13 (92.90)	10 (66.67)		
Age (year)		54.47 ± 7.13	56.29 ± 7.74	56.73 ± 5.12	*χ* ^2^ = 1.275	0.529
Heredity (%)						
Yes		11 (73.3)	8 (57.10)	7 (46.70)	*χ* ^2^ = 2.239	0.327
No		4 (26.70)	6 (42.90)	8 (53.30)		
Course of disease (month)		86.00 ± 52.30	78.93 ± 70.50	73.73 ± 45.15	*χ* ^2^ = 1.460	0.482
SBP (mmHg)		136.33 ± 17.43	134.93 ± 9.05	136.27 ± 14.16	*F* = 0.045	0.956
DBP (mmHg)		89.47 ± 8.43	86.29 ± 7.35	81.33 ± 9.16	*F* = 0.999	0.377
SF-36 $\overset{-}{X}\pm SE$	PF	88.33 ± 2.05	85.71 ± 3.39	92.67 ± 1.68	*χ* ^2^ = 3.349	0.187
	RP	70.00 ± 10.12	75.00 ± 9.81	70.00 ± 10.69	*χ* ^2^ = 0.348	0.840
	BP	77.47 ± 3.66	79.07 ± 3.97	82.20 ± 4.37	*χ* ^2^ = 1.198	0.549
	GH	54.47 ± 4.90	50.64 ± 3.76	60.53 ± 3.72	*F* = 1.414	0.255
	VT	70.33 ± 4.77	68.21 ± 4.96	81.33 ± 2.91	*F* = 2.691	0.082
	SF	85.93 ± 4.66	82.54 ± 4.15	84.44 ± 5.06	*χ* ^2^ = 0.911	0.634
	RE	84.44 ± 7.88	73.81 ± 9.36	68.89 ± 10.01	*χ* ^2^ = 1.582	0.453
	MH	69.60 ± 4.55	68.86 ± 4.00	78.00 ± 2.16	*F* = 1.897	0.163

**Table 2:** Changes of average SBP and DBP of three groups between pretreatment and posttreatment ($\overset{-}{X}\pm SD$).

Time	Items	Group A (*n* = 15)	Group B (*n* = 14)	Group C (*n* = 15)	Statistical value	*P*
BA	SBP (mmHg)	136.33 ± 17.43	134.93 ± 9.05	136.27 ± 14.16	*χ* ^2^ = 0.091	0.955
	DBP (mmHg)	89.47 ± 8.43	86.29 ± 7.35	85.33 ± 9.16	*χ* ^2^ = 2.634	0.268
AA	SBP (mmHg)	130.00 ± 13.44	129.71 ± 8.66	133.60 ± 12.86	*F* = 0.489	0.617
	DBP (mmHg)	87.87 ± 5.04	86.71 ± 6.11	85.07 ± 7.29	*F* = 0.769	0.470

BA: pretreatment; AA: posttreatment.

**Table 3:** The changes of brain regions of group A after treatment versus before treatment.

Number of voxels	Hemi	Brain areas	BA	*T* (peak intensity)	Peak MNI coordinate		
					*X*	*Y*	*Z*
1248	R	Inferior occipital gyrus	19	5.8152	39	−93	−15
313	L	Inferior frontal gyrus in triangular and opercular part	44, 45	3.6219	−9	60	18
366	L	Postcentral gyrus, inferior parietal, excluding supramarginal and angular gyri, supramarginal gyrus	3, 4	5.9703	−45	−27	42
344	R	Insula, superior temporal gyrus	21	−5.0278	45	−3	−15
551	R	Precuneus	—	−6.7036	12	−60	42
851	R	Anterior cingulate and paracingulate gyrus, median cingulate and paracingulate gyrus, medial superior frontal gyrus	8, 24, 32	−6.5526	6	12	27

**Table 4:** The changes of brain regions of group B after treatment versus before treatment.

Number of voxels	Hemi	Brain areas	BA	*T* (peak intensity)	Peak MNI coordinate		
					*X*	*Y*	*Z*
91	R	Parahippocampal gyrus, temporal pole (superior and middle temporal gyrus)	28	4.5731	24	9	−33
87	R	Precuneus	31	4.689	18	−48	36
102	L	Median cingulate and paracingulate gyrus	24	−4.5841	−3	18	33
122	R	Inferior parietal, excluding supramarginal and angular gyri, supramarginal gyrus	40	−4.1277	42	−39	45
119	L	Paracentral lobule	—	−3.717	−6	−24	48

**Table 5:** The changes of brain regions of group C after treatment versus before treatment.

Number of voxels	Hemi	Brain areas	BA	*T* (peak intensity)	Peak MNI coordinate		
					*X*	*Y*	*Z*
100	R	Postcentral gyrus	—	3.8341	30	−18	39
99	L	Frontal lobe	—	4.3742	−30	−15	39
167	L	Putamen	—	−4.1377	−21	6	12

**Table 6:** The comparison of changes of brain regions among 3 groups after treatment.

Number of voxels	Hemi	Brain areas	Brodmann area	*T* (peak intensity)	Peak MNI coordinate		
					*X*	*Y*	*Z*
Group A versus group B							
739	R	Precuneus, median cingulate and paracingulate gyrus	7, 31	4.8486	12	−48	−36
539	R	Postcentral gyrus, paracentral lobule	3, 5	−5.545	15	−39	72

Group A versus group C							
120	R	Superior and middle frontal gyrus	9, 46	3.5548	21	42	48
98	R	Medial superior frontal gyrus	10	3.6219	−9	60	18

Group B versus group C							
377	R	Middle frontal gyrus in orbital part	46	5.5729	45	51	−9
496	R	Superior and middle occipital gyrus	18, 19	−4.3424	21	−78	18
325	R	Paracentral lobule	4	−4.0411	0	−30	69
